# Development of Linear Interpolation System for SMK Model Parameters Evaluated from Cellular-Scale Simulation (LISMEC) and its application to BNCT dosimetry

**DOI:** 10.1093/jrr/rraf075

**Published:** 2026-02-05

**Authors:** Takafumi Shigehira, Tubasa Watanabe, Minoru Suzuki, Yuho Hirata, Tatsuhiko Ogawa, Atsushi Fujimura, Yoshinori Sakurai, Tatsuhiko Sato

**Affiliations:** Particle Radiation Oncology Research Center, Institute for Integrated Radiation and Nuclear Science, Kyoto University, 2-1010, Asashiro-nishi, Kumatori-cho, Sennan-gun, Osaka 590-0494, Japan; Particle Radiation Oncology Research Center, Institute for Integrated Radiation and Nuclear Science, Kyoto University, 2-1010, Asashiro-nishi, Kumatori-cho, Sennan-gun, Osaka 590-0494, Japan; Particle Radiation Oncology Research Center, Institute for Integrated Radiation and Nuclear Science, Kyoto University, 2-1010, Asashiro-nishi, Kumatori-cho, Sennan-gun, Osaka 590-0494, Japan; Research Group for Radiation Transport Analysis, Nuclear Science and Engineering Center, Japan Atomic Energy Agency (JAEA), 2-4 Shirakata, Tokai, Ibaraki 319-1195, Japan; Research Group for Radiation Transport Analysis, Nuclear Science and Engineering Center, Japan Atomic Energy Agency (JAEA), 2-4 Shirakata, Tokai, Ibaraki 319-1195, Japan; Department of Cellular Physiology, Neutron Therapy Research Center, Okayama University Graduate School of Medicine, Dentistry and Pharmaceutical Sciences, 2-5-1 Shikata-cho, Kita-ku, Okayama 700-8558, Japan; Particle Radiation Oncology Research Center, Institute for Integrated Radiation and Nuclear Science, Kyoto University, 2-1010, Asashiro-nishi, Kumatori-cho, Sennan-gun, Osaka 590-0494, Japan; Research Group for Radiation Transport Analysis, Nuclear Science and Engineering Center, Japan Atomic Energy Agency (JAEA), 2-4 Shirakata, Tokai, Ibaraki 319-1195, Japan

**Keywords:** BNCT, microdosimetry, boron distribution, cell morphology

## Abstract

Boron neutron capture therapy (BNCT) utilizes high linear energy transfer (LET) α-particles and ^7^Li ions generated through the ^10^B(n, α)^7^Li reaction. Precise dosimetry is essential for maximizing therapeutic efficacy while minimizing normal tissue adverse events, considering the microscopic distribution of ^10^B and cellular structures. Recently, the photon isoeffective dose (*D*_isoE_) has been proposed as a more appropriate metric for BNCT treatment planning and can be evaluated using the stochastic microdosimetric kinetic (SMK) model. However, clinical implementation of the SMK model remains challenging due to the difficulty of evaluating its input parameters, which requires computationally intensive radiation transport simulations at the cellular scale. To address this issue, we developed LISMEC (Linear Interpolation System for Stochastic Microdosimetric Kinetic model parameters Evaluated from Cellular-scale simulation), a rapid estimation framework based on precomputed cellular-scale PHITS (Particle and Heavy Ion Transport code System) simulations covering various cell geometries and boron distributions. By applying a linear interpolation algorithm, LISMEC enables the retrieval of SMK model parameters without the need for computationally intensive cellular-scale simulations. The utility of LISMEC, in conjunction with PHITS, was demonstrated through simulations of various irradiation scenarios in reactor-based BNCT. The results showed that *D*_isoE_ values ranged from 7.4 to 32.7 Gy, even under a fixed macroscopic ^10^B concentration of 60 ppm. These findings emphasize the importance of incorporating a microscopic distribution of ^10^B and cellular structures into BNCT treatment planning.

## INTRODUCTION

Boron neutron capture therapy (BNCT) is a radiation modality that leverages high linear energy transfer (LET) alpha particles and ^7^Li ions emitted via the ^10^B(n, α)^7^Li reaction [1]. Because these charged particles have an extremely short range—<10 μm—they selectively deposit substantial energy within tumor cells, producing a potent cytotoxic effect. In 2020, BNCT for unresectable head and neck cancers obtained national health insurance approval in Japan [2, 3]. This approval has encouraged research and clinical studies aimed at expanding the types of diseases treatable with BNCT. Drugs containing boron that are currently available have limited applicability to certain tumors. Therefore, developing novel boron-containing agents is essential to realize broader clinical applications [4]. A commonly used metric for comparing different radiation types is the relative biological effectiveness (RBE), defined as the ratio of doses required to achieve the same biological endpoint, typically benchmarked against X-rays [5, 6]. In BNCT, however, an additional factor called the compound biological effectiveness (CBE) has been introduced to reflect the in-cell behavior of boron agents [7–10]. Although CBE values incorporate parameters such as boron compound type, biological endpoints and cell characteristics, they often rely on empirical assumptions and may insufficiently address dose dependence, synergistic effects among multiple radiation components or the highly localized nature of dose distributions [11–14].

To overcome these limitations, the photon isoeffective dose (*D*_isoE_) has been proposed as a more theoretically grounded index [15, 16]. However, these studies cannot explicitly consider cell morphology and intracellular ^10^B distribution. Then, we previously developed a method to apply the stochastic microdosimetric kinetic (SMK) model [17, 18] to BNCT dosimetry, enabling the direct calculation of *D*_isoE_ [13, 19] while taking these effects into consideration. The SMK model predicts cell survival by evaluating the probability distributions of energy deposition in both the cell nucleus and subnuclear domains—an approach well suited for high-LET therapies like BNCT, carbon-ion therapy [20] and targeted alpha therapy [21], which exhibit significant dose heterogeneity at microscopic scales.

Nevertheless, harnessing the SMK model for BNCT requires detailed cellular-scale Monte Carlo simulations that account for cell morphology and intracellular ^10^B distribution, followed by the computation of *D*_isoE_. Constructing specialized cell models for each boron agent or clinical scenario, and then running resource-intensive simulations, has hindered the broader adoption of this approach.

In this study, we performed comprehensive cellular-scale simulations under various conditions using the general-purpose Particle and Heavy Ion Transport code System (PHITS) [22]. From these precomputed results, we developed an Excel-based system—termed ‘LISMEC’ (Linear Interpolation system for Stochastic Microdosimetric kinetic model parameters Evaluated from Cellular-scale simulation)—that permits rapid and straightforward estimation of SMK model parameters. With LISMEC, users only need to specify seven key parameters related to cell morphology, ^10^B distribution and microdosimetric properties to obtain *D*_isoE_ over a wide range of conditions without additional complex simulations.

In this article, we detail the development and implementation of LISMEC and demonstrate its utility for BNCT dosimetry by applying it to radiation field data from the Kyoto University Reactor (KUR) at the Institute for Integrated Radiation and Nuclear Science. Specifically, we compute *D*_isoE_ for diverse cellular structures and boron distributions, and then analyze the resultant dose profiles. Finally, we discuss challenges for clinical application and propose directions for future work.

## MATERIALS AND METHODS

### Procedure for calculating *D*_isoE_ using the SMK model

**Fig. 1:**
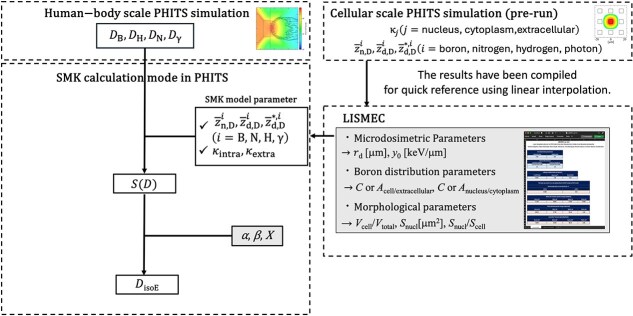
Flowchart for calculating *D*_isoE_ developed in this study.

Figure 1 provides an overview of the workflow for calculating *D*_isoE_ in this study. Parameters shown in gray boxes represent user inputs. The process consists of the following steps:

1. Human-body-scale PHITS simulation:
  In this step, a macroscale PHITS simulation is conducted to calculate the absorbed doses for four BNCT dose components: boron, nitrogen, hydrogen and photon, respectively [23, 24].
2. Retrieval of SMK model parameters via LISMEC:
  Building on the results of a precomputed ‘cellular-scale PHITS simulation’, LISMEC provides the SMK model parameters for the specified cell morphology and ^10^B distribution.
3. Calculation of the cell survival fraction *S* (*D*):
  Using the dose data from steps 1 and 2, the cell survival fraction *S* (*D*) is determined.
4. Calculation of *D*_isoE_:
  Finally, given user-specified values for *α* (Gy^−1^), *β* (Gy^−2^) (the reference radiation’s linear and quadratic parameters) and *X* (Gy per fraction), the photon isoeffective dose *D*_isoE_ is calculated.

### Input parameters for the SMK model

The following parameters must be provided to the SMK model:

-
${\kappa}_{\mathrm{intra}},{\kappa}_{\mathrm{extra}}$ : conversion factors that translate the macroscopic boron dose into the cell nuclear dose [25]. ${\kappa}_{\mathrm{intra}}$ applies to ^10^B located inside the cell, whereas ${\kappa}_{\mathrm{extra}}$ applies to ^10^B outside the cell.
-
${\overline{z}}_{\mathrm{n},\mathrm{D}}^i\ \left(i=\mathrm{B},\mathrm{N},\mathrm{H},\mathrm{\gamma} \right)$ : the dose-mean cell-nucleus dose for each dose component *i*.
-
${\overline{z}}_{\mathrm{d},\mathrm{D}}^i\left(i=\mathrm{B},\mathrm{N},\mathrm{H},\mathrm{\gamma} \right)$ : the dose-mean domain dose within the cell nucleus.
-
${\overline{z}}_{\mathrm{d},\mathrm{D}}^{\ast, i}\left(i=\mathrm{B},\mathrm{N},\mathrm{H},\mathrm{\gamma} \right)$ : the saturation-corrected domain dose, accounting for the overkill effect.

In this study, we precomputed these parameters across various cell morphologies and ^10^B distributions, then incorporated them into an Excel-based system (LISMEC) where they can be efficiently retrieved via linear interpolation. Consequently, when clinical conditions or boron agents change, complex cellular-scale simulations need not be repeated. *D*_isoE_ can thus be computed promptly and with minimal computational burden using the SMK model.

### Cellular-scale PHITS simulation

In this study, we constructed 512 cell models by systematically varying three parameters that characterize the cellular morphology: (1) the nuclear surface area (*S*_nucl_), (2) the ratio of nuclear to total cell surface area (*S*_nucl_/*S*_cell_) and (3) the fraction of total volume occupied by cells (*V*_cell_/*V*_total_). Each model comprises an 11 × 11 × 11 array of cubic cells surrounded by an extracellular region. Specifically, we set (*S*_nucl_) to 9, 36, 81 or 144 μm^2^; *S*_nucl_/*S*_cell_ to 0.1, 0.2, 0.3, 0.4, 0.5, 0.6, 0.7 or 0.8; and *V*_cell_/*V*_total_ to ~0.3–0.95 by adjusting the intercellular distance (0.1, 0.5, 1, 2, 3, 4, 5, 6, 7, 8, 9, 10, 11, 12, 13 and 14 μm). All the regions were assigned a density of 1 g cm^−3^ and an elemental composition of 10.7% hydrogen, 12.1% carbon, 2% nitrogen, 71.4% oxygen [26]. The remaining 3.8% other elements was neglected in the calculations, resulting in a total of 96.2%.

For each of the 512 models, we considered three scenarios for the ^10^B(n, α)^7^Li reaction: (1) in the extracellular space, (2) in the cytoplasm or (3) in the nucleus. Radiation transport calculations were then performed using PHITS versions 3.33 and 3.341.

**Fig. 2:**
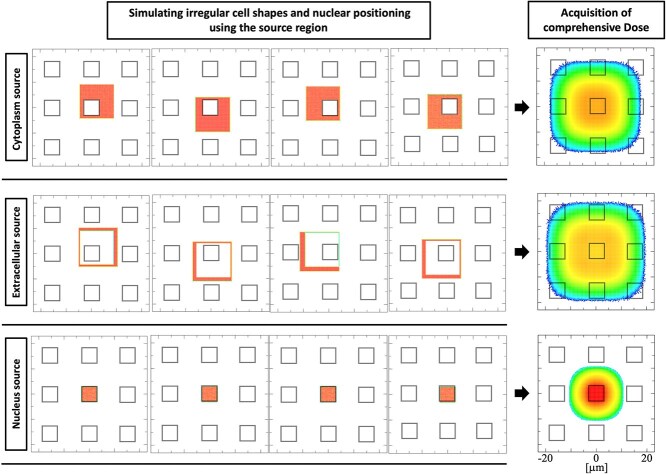
Illustration of the source regions and the overall dose distribution for the extracellular region, cytoplasm, and nucleus.

Figure 2 illustrates the source-generation procedure. In the nucleus scenario, a random reaction point was sampled within the nucleus, emitting alpha particles and ^7^Li ions via a correlated source function. The correlated source function is a built-in feature of PHITS enabling simultaneous sampling of multiple radiations from the same spatial coordinate. In this study, we utilized isocorr = 3. For cytoplasmic and extracellular scenarios, we accounted for spatial configuration by randomizing the relative positions of the nucleus and the source region in each history (Fig. 2). Since the default PHITS capabilities cannot accommodate this, we employed a user-defined source function. The ^10^B(*n*, α)^7^Li reaction proceeds via two branching channels, one accompanied by gamma emission and one without, each yielding alpha particles and ^7^Li ions of different energies. We conducted separate simulations for each channel and combined them according to their branching ratios.

In Fig. 2, the nine small squares represent the cell nucleus, where dose calculations are performed. Each of the four images within each scenario shows an example of one possible arrangement. The dose distribution on the right is obtained by aggregating many such randomized arrangements, including the four cases illustrated here.

To model the dose by neutron-induced reactions by nitrogen and hydrogen, as well as the gamma-rays produced by these reactions, we uniformly generated source particles from cells and interstitial regions in the center of the cell model. The source information was derived from a macroscale simulation of a 1 cm^3^ International Commission on Radiation Units and Measurements (ICRU) phantom under neutron irradiation [13]. All the particles were transported down to 1 keV, and electrons were managed using the Electron Gamma Shower (EGS) mode [27]. We selected a sufficient number of histories to keep the statistical uncertainty < 1% in each ‘bin’. Here, a ‘bin’ is a small interval of energy or dose used to categorize simulation results for statistical analysis. From these simulations, we extracted the mean energy deposited in the cell nuclei and in the entire cell (nuclei, cytoplasm and extracellular space). We also tallied the frequency distributions of specific energies (nuclear and domain). To calculate domain-specific energies, we used PHITS’s microdosimetric function [t-sed] tally [14] with eight domain diameters: 0.05, 0.1, 0.2, 0.3, 0.4, 0.5, 0.7 and 1 μm.

### Development of LISMEC

#### Outline of the system

**Fig. 3:**
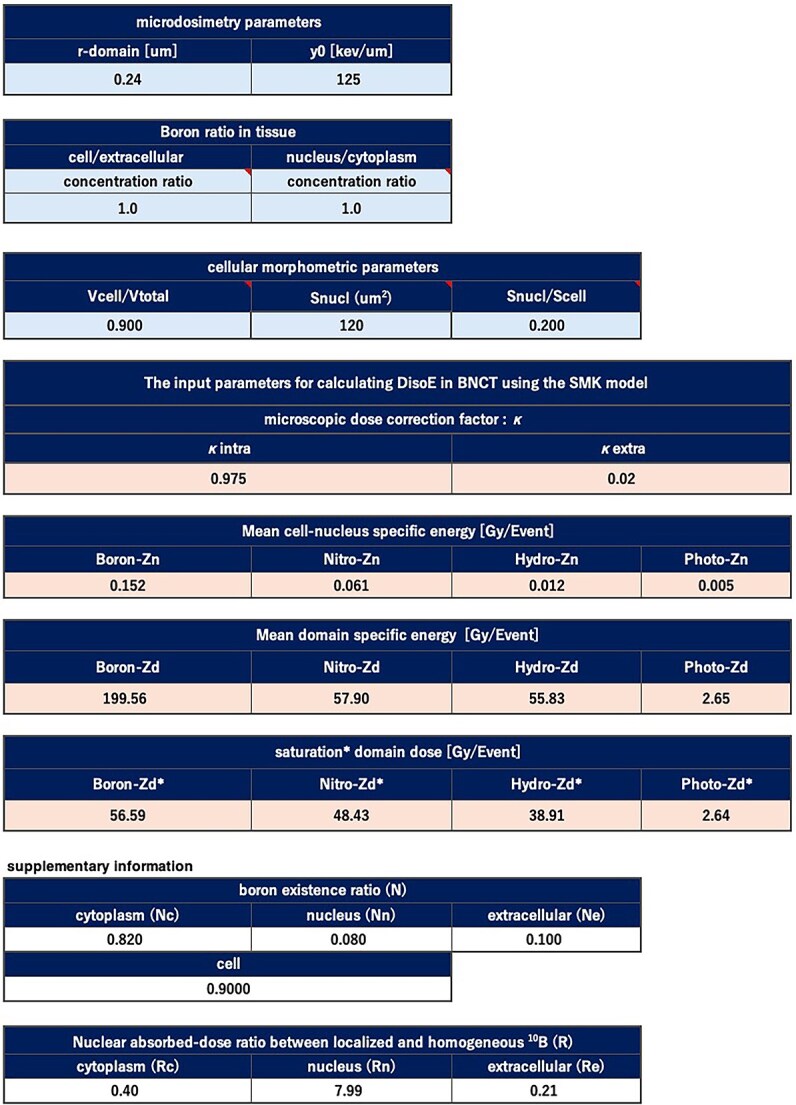
User interface of the LISMEC system.

To streamline the retrieval of ${\kappa}_{\mathrm{j}}\left(\mathrm{j}=\mathrm{nucleus},\mathrm{cytoplasm},\mathrm{extracellular}\right) \, {\overline{z}}_{\mathrm{n},\mathrm{D}}^i,{\overline{z}}_{\mathrm{d},\mathrm{D}}^i,{\overline{z}}_{\mathrm{d},\mathrm{D}}^{\ast, i}\left(i=\mathrm{B},\mathrm{N},\mathrm{H},\mathrm{\gamma} \right)$—the parameters required for SMK model calculations under any cell morphology or ^10^B distribution—we developed an Excel-based system called ‘LISMEC’. This system applies linear interpolation to the precomputed simulation data described in the previous section. The user interface is shown in Fig. 3.

By specifying inputs in three categories—(1) SMK model parameters, (2) boron distribution parameters and (3) cellular morphologic parameters—LISMEC yields the ‘boron dose correction coefficient’, ‘dose-mean cell-nucleus specific energy’, ‘dose-mean domain-specific energy’ and ‘saturation-corrected dose-mean domain-specific energy’ for each BNCT dose component. Note that specific energy is defined as the quotient of the energy imparted to a target volume by its mass [28]. Although it shares the same units as the absorbed dose, it is a stochastic quantity that should be expressed by its probability density. In Fig. 3, the pale blue fields represent user inputs, while the pale red fields show the outputs. If certain parameters are unknown or difficult to measure, default values can be substituted.

Furthermore, LISMEC automatically arranges these outputs on a separate Excel sheet in a format compatible with PHITS. By simply copying and pasting the generated data into a PHITS input file, users can seamlessly incorporate the LISMEC-derived parameters for *D*_isoE_ calculations.

#### Input parameters of LISMEC

The input parameters in LISMEC (Fig. 3) are designed to capture morphological features and boron distribution data that closely reflect actual cellular conditions. By integrating experimentally measured or literature-based morphological characteristics and boron distribution profiles, BNCT dose assessments can more accurately reflect real biological scenarios.

##### Domain radius: r_d_ (mm)

This parameter represents the domain radius in the MK model. Initially, it was defined as the distance within the cell nucleus over which DNA damage can diffuse. In practice, it is typically determined by least-squares fitting of cell survival data obtained from multiple radiation types.

##### Saturation parameter: y_0_ (keV mm^−1^)

This parameter sets the threshold for the overkill effect, in which cells receive more energy than necessary to induce cell death. As with *r*_d_, it is typically determined by least-squares fitting of cell survival data from multiple radiation types.

##### Boron uptake ratio into cell: C_cell_/C_extra_ or A_cell_/A_extra_

This parameter reflects the extent of boron uptake by cells relative to the extracellular space. It can be expressed either as a concentration ratio (*C*_cell_*/C*_extra_) or an abundance ratio (*A*_cell_*/A*_extra_). These values can be determined via immunofluorescence imaging [29] or by combining CR-39 track-etch and H&E-stained imaging [30, 31].

##### Boron uptake ratio into cell nucleus: C_nucl_/C_cyto_ or A_nucl_/A_cyto_

This parameter reflects the extent of boron accumulation in the nucleus relative to the cytoplasm. It can be expressed as either a concentration ratio (*C*_cell_*/C*_extra_) or an abundance ratio (*A*_cell_*/A*_extra_). These values are also typically determined via immunofluorescence imaging [29] or by combining CR-39 track-etch with Hematoxylin Eosin (H&E)-stained imaging [30, 31].

##### Volume ratio of cells to total tissue: V_cell_/V_total_

This parameter indicates the fraction of the total tissue volume occupied by cells. It can be determined through immunofluorescence or H&E-stained image analysis [29], as well as by MRI-based methods [32–34].

##### Mean cross-sectional area of cell nucleus and its ratio to cell: S_nucl_ (mm^2^) and S_nucl_/S_cell_

These parameters represent the average cross-sectional area of the nucleus (*S*_nucl_ [μm^2^]) and the fraction of the cell’s total cross-sectional area occupied by the nucleus (*S*_nucl_/*S*_cell_). They can be measured via immunofluorescence or H&E-stained image analysis [11, 29, 35].

#### Output parameters of LISMEC

##### Conversion factor from the macroscopic boron dose to cell nucleus dose: κ_intra_ and κ_extra_

In the SMK model, the cell survival fraction is estimated based on the dose delivered to the cell nucleus. Hence, a conversion factor κ is required to translate the macroscale kerma into the cell-nucleus dose. In this study, κ is defined as follows:


(1) \begin{align*} \kappa = N_{\mathrm{n}} R_{\mathrm{n}} + N_{\mathrm{c}} R_{\mathrm{c}} + N_{\mathrm{e}} R_{\mathrm{e}}, \end{align*}

where *N*_n_, *N*_c_ and *N*_e_ are the relative proportions of ^10^B in the nucleus, cytoplasm and extracellular space, respectively. Meanwhile, *R*_n_, *R*_c_ and *R*_e_ represent the ratio of the cell-nucleus dose when ^10^B is localized in the nucleus, cytoplasm or extracellular space, respectively, to the cell-nucleus dose with uniformly distributed ^10^B. The total proportion of ^10^B across all compartments is normalized to 1.0:


(2) \begin{align*} N_{\mathrm{n}} + N_{\mathrm{c}} + N_{\mathrm{e}} = 1.0 \end{align*}

Furthermore,


(3) \begin{align*} \kappa_{\mathrm{intra}}= N_{\mathrm{n}} R_{\mathrm{n}} + N_{\mathrm{c}} R_{\mathrm{c}},\end{align*}

(4) \begin{align*}\kappa_{\mathrm{extra}}= N_{\mathrm{e}} R_{\mathrm{e}} . \end{align*}

By these definitions, Equation (1) can be rewritten as


(5) \begin{align*} \kappa = \kappa_{\mathrm{intra}} + \kappa_{\mathrm{extra}}. \end{align*}

##### Dose-mean cell-nucleus specific energy is ${\overline{z}}_{\mathrm{n},\mathrm{D}}^i\left(i=\mathrm{B},\mathrm{N},\mathrm{H},\mathrm{\gamma} \right)$

Using the [t-deposit] function in the cellular-scale PHITS simulation, we obtain the probability density ${f}_{\mathrm{n},1}\left({z}_{\mathrm{n}}\right)$ for the specific energy ${z}_{\mathrm{n},\mathrm{D}}$ imparted by each radiation component $\left(\mathrm{B},\mathrm{N},\mathrm{H},\mathrm{\gamma} \right)$ to the cell nucleus. From this distribution, we then calculate the dose-mean cell-nucleus specific energy ${\overline{z}}_{\mathrm{n},\mathrm{D}}^i\ \left(i=\mathrm{B},\mathrm{N},\mathrm{H},\mathrm{\gamma} \right)$according to equation (6).


(6) \begin{align*} {\overline{z}}_{\mathrm{n},\mathrm{D}}={\int}_0^{\infty }{z}_{\mathrm{n}}{f}_{\mathrm{n},1}\left({z}_{\mathrm{n}}\right)\;\mathrm{d}{z}_{\mathrm{n}}\kern0.5em \end{align*}

##### *Dose-mean and saturation-corrected domain specific energy:*  ${\overline{z}}_{\mathrm{d},\mathrm{D}}^i$*and*  ${\overline{z}}_{\mathrm{d},\mathrm{D}}^{\ast, i}\\ \left(i=\mathrm{B},\mathrm{N},\mathrm{H},\mathrm{\gamma} \right)$

Using the microdosimetric function in the cellular-scale PHITS simulation, we obtained the probability density ${f}_{\mathrm{d},1}\left({z}_d\right)$ for the specific energy imparted by each radiation component $\left(\mathrm{B},\mathrm{N},\mathrm{H},\mathrm{\gamma} \right)$ within the domain. From this distribution, we then calculated the dose-mean and saturation-corrected domain-specific energies ${\overline{\mathrm{z}}}_{\mathrm{d},\mathrm{D}}^{\mathrm{i}}$and ${\overline{\mathrm{z}}}_{\mathrm{d},\mathrm{D}}^{\ast, \mathrm{i}}\ \left(i=\mathrm{B},\mathrm{N},\mathrm{H},\mathrm{\gamma} \right)$ according to Equations (7) and (8).


(7) \begin{equation*} {\overline{z}}_{\mathrm{d},\mathrm{D}}=\frac{\int_0^{\infty }{z}_{\mathrm{d}}^2{f}_{\mathrm{d},1}\left({z}_{\mathrm{d}}\right)\;d{z}_{\mathrm{d}}}{\int_0^{\infty }{z}_{\mathrm{d}}{f}_{\mathrm{d},1}\left({z}_{\mathrm{d}}\right)\;d{z}_{\mathrm{d}}}. \end{equation*}

(8) \begin{equation*} {\overline{z}}_{\mathrm{d},\mathrm{D}}^{\ast }=\frac{z_0^2{\int}_0^{\infty}\left\{1-\exp \left(-{\left(\frac{z_{\mathrm{d}}}{z_0}\right)}^2\right)\right\}{f}_{\mathrm{d},1}\left({z}_{\mathrm{d}}\right)\;\mathrm{d}{z}_{\mathrm{d}}}{{\overline{z}}_{\mathrm{d},F}} \end{equation*}

In this study, we treated ${\overline{z}}_{\mathrm{d},\mathrm{D}}^{\ast }$ (with a saturation parameter *y*_0_ = 1 × 10^4^ [keV mm^−1^]) as equivalent to ${\overline{z}}_{\mathrm{d},\mathrm{D}}$.

We then compiled the cellular-scale PHITS simulation results in Excel and used the VLOOKUP, INDEX and MATCH functions to perform linear interpolation, enabling efficient retrieval of SMK model parameters for arbitrary conditions.

#### Validation of the interpolation method accuracy

We assessed the accuracy of the linear interpolation method in two steps. First, under a uniform boron distribution (boron concentration ratio in tissue: cell/extracellular = 1, nucleus/cytoplasm = 1), κ_all_ is theoretically equal to 1. We used this as our accuracy benchmark. Second, we quantified the deviation of κ_all_ from 1 at linearly interpolated locations within the parameter space. In nonuniform conditions, we conducted several validation simulations (four points) for scenarios that had not been evaluated during LISMEC’s development and compared the outcomes with LISMEC’s linear-interpolation results.

### Applications of LISMEC

#### Sensitivity analysis of SMK model parameters using LISMEC

We used LISMEC to investigate how the boron uptake ratio (*C*_cell_*/C*_extra_) and cellular morphometric parameters (*V*_cell_/*V*_total_, *S*_nucl_*/S*_cell_ and *S*_nucl_) affect the SMK model parameters. We varied four parameters (*C*_cell_*/C*_extra_, *V*_cell_/V_total_, *S*_nucl_*/S*_cell_ and *S*_nucl_) and evaluated the resulting values of κ$, {\overline{z}}_{\mathrm{n},\mathrm{D}}^{\mathrm{B}},\kern0.5em {\overline{z}}_{\mathrm{d},\mathrm{D}}^{\mathrm{B}}\kern0.5em \mathrm{and}\kern0.50em {\overline{z}}_{\mathrm{d},\mathrm{D}}^{\ast, \mathrm{B}}$. The sensitivity analysis was performed under the following conditions: ・*S*_nucl_/*S*_cell_ = 0.2, 0.8 ・*V*_cell_/*V*_total_ = 0.3, 0.9 ・*C*_cell_*/C*_extra_ = 0, 0.1, 0.5, 1, 3.5 ・*S*_nucl_ = 60, 120 mm^2^. We assumed a uniform boron concentration inside the cell (*C*_nucl_/*C*_cyto_ = 1) [36]. Reference values for the nuclear cross-sectional area were drawn from basal cell carcinoma (65 μm^2^ [37]) and breast carcinoma (150 μm^2^ [38]). The upper limit of 3.5 for the boron uptake ratio was adopted from a previous study [39] as a representative example.

#### D_isoE_ evaluation in BNCT radiation fields

**Fig. 4:**
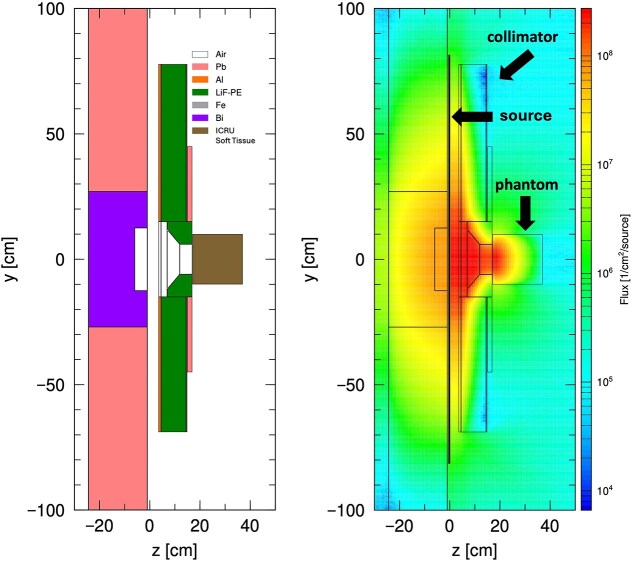
KUR–HWNIF radiation field. (Left) Cross-sectional view of the computational geometry representing the KUR irradiation port and the phantom. (Right) Neutron fluence distribution obtained via a PHITS simulation of the same geometry under the KUR thermal–epithermal neutron mode (CO0000F). The color scale indicates the magnitude of the neutron fluence.

In this study, we employed the epithermal neutron irradiation mode (CO-0000-F mode) of the Heavy Water Neutron Irradiation Facility (HWNIF) installed at Kyoto University Reactor (KUR) as our BNCT radiation field [40, 41]. Figure 4 illustrates the computational setup used in the simulation and shows the fluence distribution obtained via PHITS. The PHITS simulations were conducted under the following conditions:

· Irradiation geometry: Neutrons were directed from a 12-cm-diameter beam port onto a cubic phantom measuring 20 × 20 × 20 cm^3^.

· Phantom: Composed of ICRU soft tissue enriched with 60 ppm of ^10^B [42, 43].

· Calculation: Using the [t-track] tally, we calculated absorbed doses for each BNCT component from the neutron fluence using the kerma approximation at 1 cm intervals from the phantom’s surface to a depth of 20 cm. We ran 20 000 000 histories to keep the statistical uncertainty <1%.

Using these results, we converted the absorbed doses into *D*_isoE_ under various conditions specified in Section 2.5.1 (Sensitivity Analysis of the SMK Model Parameters Using LISMEC) and evaluated the resulting *D*_isoE_ (Fig. 6).

We also compared *D*_isoE_ with the CBE- and RBE-weighted doses, as well as the absorbed dose (Fig. 7). For the *D*_isoE_ calculations in Fig. 7, we adopted: ・*C*_cell_*/C*_extra_ = 1 ・*S*_nucl_/*S*_cell_ = 0.2 ・*V*_cell_/*V*_total_ = 0.9 ・*S*_nucl_ = 120 μm^2^. These settings were chosen simply to illustrate how *D*_isoE_ can be evaluated in a BNCT radiation field. Specifically, *C*_cell_/*C*_extra_ = 1 minimizes the influence of the other parameters (*V*_cell_/*V*_total_, *S*_nucl_/*S*_cell_, *S*_nucl_), so these particular values are not intended to have any special significance.

For the CBE- and RBE-weighted dose calculations in Fig. 7, we assigned CBE and RBE values of 3.8, 2.5, 2.5 and 2.5 to ^10^B, N, H and γ, respectively [13]. Because the absolute absorbed dose is critical for evaluating *D*_isoE_, we assumed a blood boron concentration of 20 ppm [42] and a skin-to-blood ratio of 1.1 [44], resulting in 22 ppm in the skin. We then adjusted the normalization so that the CBE- and RBE-weighted dose at the skin reached 12 Gy-Eq [9]. At this point, we set CBE and RBE for ^10^B, N, H and γ at 2.5, 2.5, 2.5 and 1, respectively [13]. These choices ensured proper scaling of the absolute dose under realistic BNCT irradiation conditions. Furthermore, we employed the following SMK model parameters:

·*r*_0_ = 0.24 [μm] [13, 19] ·*y*_0_ = 125 [keV μm^−1^] [28] ·*α*_0_ = 0.0422 [Gy^−1^] [13, 19].

·*β*_0_ = 0.00822 [Gy^−2^] [13, 19] ·*α =* 0.0633 [Gy^−1^] [13, 19] ·*β* = 0.00822 [Gy^−2^] [13, 19].

Except for *y*_0_ [keV μm^−1^], these parameters were derived from survival data for SCC VII squamous cell carcinomas [13, 19, 45].

## RESULTS

### Sensitivity analysis of SMK model parameters using LISMEC

**Fig. 5:**
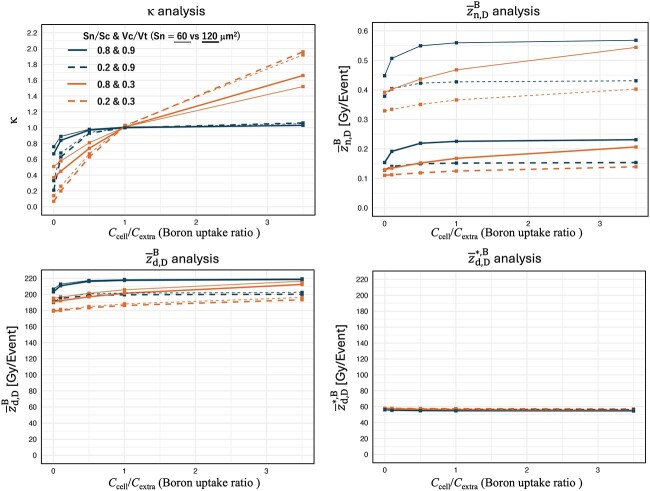
SMK model parameter analysis using LISMEC.

Figure 5 illustrates the analysis results for κ as well as ${\overline{z}}_{\mathrm{n},\mathrm{D}}^{\mathrm{B}},{\overline{z}}_{\mathrm{d},\mathrm{D}}^{\mathrm{B}}\ \mathrm{and}\ {\overline{z}}_{\mathrm{d},\mathrm{D}}^{\ast, \mathrm{B}}$. On the horizontal axis, we plotted the boron uptake ratio *C*_cell_/*C*_extra_, while the vertical axis displays κ, ${\overline{z}}_{\mathrm{n},\mathrm{D}}^{\mathrm{B}},\kern0.5em {\overline{z}}_{\mathrm{d},\mathrm{D}}^{\mathrm{B}}$ and ${\overline{z}}_{\mathrm{d},\mathrm{D}}^{\ast, \mathrm{B}}$. These results compare how changes in cellular morphologic parameters (*V*_cell_/*V*_total_, *S*_nucl_/*S*_cell_ and *S*_nucl_) and the boron uptake ratio (*C*_cell_/*C*_extra_) affect these quantities. From Fig. 5, it is evident that κ and ${\overline{z}}_{\mathrm{n},\mathrm{D}}^{\mathrm{B}}$ strongly depend on the boron uptake ratio and cellular morphologic parameters. In contrast, ${\overline{z}}_{\mathrm{d},\mathrm{D}}^{\mathrm{B}}$ and ${\overline{z}}_{\mathrm{d},\mathrm{D}}^{\ast, \mathrm{B}}$show only minor dependence on those same factors.

### *D*_isoE_ evaluation in BNCT radiation fields

**Fig. 6:**
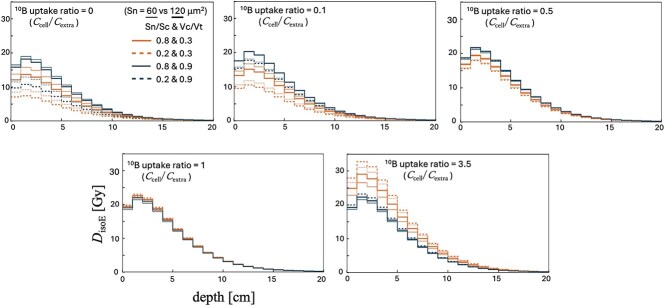
*D*  _isoE_ evaluation using LISMEC.

Figure 6 shows the depth-dose distributions of *D*_isoE_ when varying the cellular morphologic parameters (*V*_cell_/*V*_total,_  *S*_nucl_/*S*_cell_, *S*_nucl_) and the boron uptake ratio (*C*_cell_/*C*_extra_). Even under the same macro boron concentration (60 ppm), these parameter settings can substantially alter the *D*_isoE_. The lower-left panel of Fig. 6 (^10^B uptake ratio = 1) can also be regarded as the result of a *D*_isoE_ calculation that neglects both the boron distribution and the cellular structure. If the boron distribution and cell structure are not taken into account, all the other calculation results would likewise be homogenized and evaluated as equivalent to the lower-left panel of Fig. 6 (^10^B uptake ratio = 1). However, by considering the boron distribution and the cellular structure, the dose under each condition can be separated and evaluated.

**Fig. 7:**
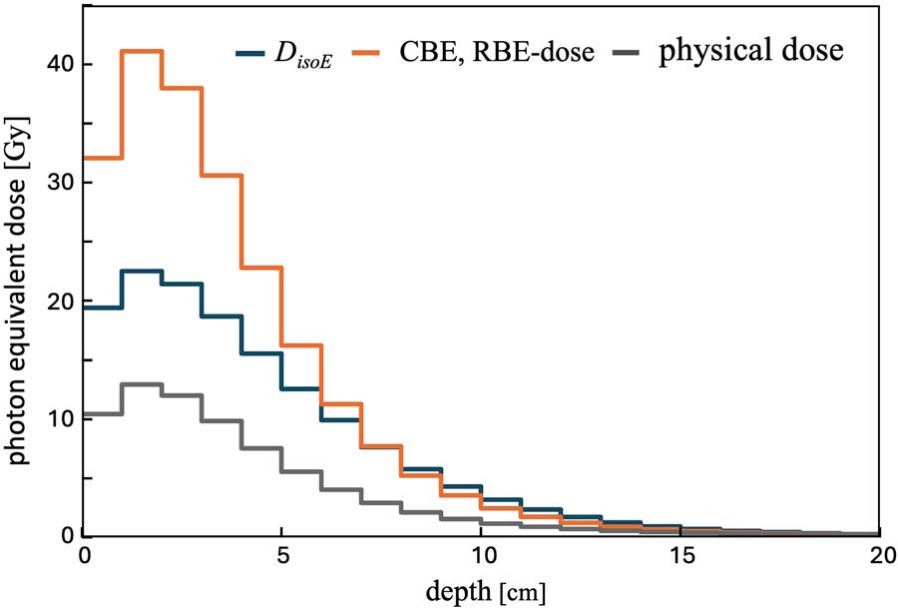
Comparison of *D*_isoE_, CBE–RBE-weighted dose and absorbed dose.

Meanwhile, Fig. 7 compares *D*_isoE_ with both the CBE- and RBE-weighted dose and absorbed dose. In regions of high dose, the CBE- and RBE-weighted doses tend to be significantly greater than the *D*_isoE_.

### Validation result of the interpolation method accuracy

We confirmed that, overall, the error was within ~+5%, and that in the region *S*_nucl_/*S*_cell_ = 0.1–0.2 it was within +15%. Comparing the computed results for conditions not evaluated during LISMEC’s development with LISMEC’s estimates, we confirmed that the error was generally within +5%. However, for nuclear localization in the *S*_nucl_/*S*_cell_ range of 0.1–0.2, an error of +21% was observed. In contrast, for cytoplasmic and extracellular localization within the same range, the errors were within 5%.

## DISCUSSION

### Sensitivity of SMK model parameters to boron uptake ratio and cellular morphologic parameters

In Fig. 5, we showed how microscopic parameters—cell occupancy (*V*_cell_/*V*_total_), the ratio of nuclear to cellular cross-sectional area (*S*_nucl_/*S*_cell_), nuclear cross-sectional area (*S*_nucl_) and boron uptake ratio (*C*_cell_/*C*_extra_)—affect the conversion factor κ (which translates macroscale kerma to cell-nucleus dose) as well as ${\overline{z}}_{\mathrm{n},\mathrm{D}}^{\mathrm{B}},{\overline{z}}_{\mathrm{d},\mathrm{D}}^{\mathrm{B}}\kern0.75em \mathrm{and}\kern0.50em {\overline{z}}_{\mathrm{d},\mathrm{D}}^{\ast, \mathrm{B}}$. The findings indicate that these microscopic parameters significantly influence the behavior of κ.

First, κ approached 1 when *C*_cell_/*C*_extra_ = 1, regardless of the cell occupancy (*V*_cell_/*V*_total_), the ratio of nuclear to cellular cross-section area (*S*_nucl_/*S*_cell_) and nuclear cross-section area (*S*_nucl_). This likely occurs because the intracellular and extracellular boron concentrations become equal, minimizing differences in boron dose between the macro and microscales.

Second, when cell occupancy is low (*V*_cell_/*V*_total_ is small), κ tends to increase in proportion to *C*_cell_/*C*_extra_. This can be interpreted as the larger extracellular space rendering a high uptake ratio more impactful, effectively concentrating boron within cells. On the other hand, when cell occupancy is high (*V*_cell_/*V*_total_ is large), κ saturates at a comparatively early stage even with an elevated uptake ratio, likely because most of the tissue is composed of cells, so the boron distribution becomes nearly homogeneous at the microscopic level, reducing discrepancies in dose evaluation.

Third, the effect of *S*_nucl_/*S*_cell_ is particularly evident for *C*_cell_/*C*_extra_ < 1, where higher values of *S*_nucl_/*S*_cell_ correlate with higher κ. This is presumably because charged particles originating from extracellular boron more readily reach the nucleus due to geometric considerations. In contrast, when *C*_cell_/*C*_extra_ > 1 and the cell occupancy is high, *S*_nucl_/*S*_cell_ has little influence because the tissue is occupied by cells. On the other hand, when *C*_cell_/*C*_extra_ > 1 and the cell occupancy is low, a smaller *S*_nucl_/*S*_cell_ ratio tended to result in a higher cell-nucleus dose. One possible explanation is that reducing *S*_nucl_/*S*_cell_ increases the relative cytoplasmic volume, thereby placing more boron within the range of BNCT-generated charged particles that reach the nucleus.

### *D*_isoE_ evaluation

Based on the mechanisms discussed in the previous section (4.1, Sensitivity of SMK Model Parameters to Boron Uptake Ratio and Cellular Morphologic Parameters), we evaluated the actual *D*_isoE_ (Fig. 6) and observed a trend similar to the changes in κ shown in Fig. 5. This suggests that κ is the dominant factor influencing *D*_isoE_.

For instance, at a depth of 2 cm, *D*_isoE_ values span ~7.4–19.0 Gy (*C*_cell_/*C*_extra_ = 0), 10.5–20.3 Gy (*C*_cell_/*C*_extra_ = 0.1), 17.9–21.7 Gy (*C*_cell_/*C*_extra_ = 0.5), 22.0–23.0 Gy (*C*_cell_/*C*_extra_ = 1) and 21.6–32.7 Gy (*C*_cell_/*C*_extra_ = 3.5).

Comprehensively, even with the same macroscale boron concentration (60 ppm), the estimated *D*_isoE_ can vary widely between ~7.4 and 32.7 Gy depending on the microscopic parameters. This wide range highlights the significance of microscopic factors that cannot be captured by evaluations based solely on macroscale concentrations.

### Limitations and future work

It is important to emphasize that the conclusions of this study were drawn under the assumption that a macroscopic boron concentration has already been observed and remains fixed. So, our findings do not suggest that increasing the uptake ratio itself lacks efficacy.

Our cell model is simple compared to actual structures. In reality, cell systems are much more complex, incorporating necrotic regions and vascular networks. A mesh-type cell model could be desirable in future system developments to better represent these complexities [46]. On the other hand, making the model overly complex significantly increases the workload, so it is important to consider a balance between the detail of the model and the effort required to build and use it.

In this study, the dosimetric evaluation is conducted using a homogeneous cell model constructed on the basis of cellular structural parameters. However, actual tumor tissue is heterogeneous: cellular structures vary by location within the tumor, and cells with diverse morphologies are mixed. Therefore, this model which uniformly assumes an identical cell shape for all cells may not faithfully reflect real conditions. This is a limitation of models that rely on simplifications.

Based on the validation results for the accuracy of the interpolation method, it should be noted that when using LISMEC, especially for *S*_nucl/_S_cell_ = 0.1–0.2, the error is +15%∽21%. The increased error in the *S*_nucl/_S_cell_ range of 0.1–0.2 was considered to be largely attributable to the linear interpolation of the nuclear-localization results.

The findings revealed in this study emphasize that, in BNCT, dose assessments can vary significantly not only with changes in macroscopic boron concentration, but also with boron distribution at the cellular level and morphological features. However, to apply these insights in clinical practice, it will be necessary to develop methods for accurately measuring and estimating microscale boron dynamics and cellular morphological parameters.

Toward clinical implementation, we will first validate the concordance between experimentally measured survival rates and LISMEC predictions. Once confirmed, we will enhance usability. At present, the parameters determined by LISMEC are applied uniformly across the entire dose distribution; in future iterations, we aim to enable region-specific parameterization. In the future, we aim to support not only tumor assessment but also the evaluation of radiation-induced adverse events in normal tissues.

In experimental validation, we plan to conduct *in vitro* studies. Although *in vivo* experiments more closely approximate actual conditions, it is difficult to impose the controlled conditions required to test the validity of our system parameters. Therefore, we chose an *in vitro* design. Validation will be based on the concordance between the surviving fraction predicted by LISMEC and that measured in irradiation experiments. To assess the boron-distribution parameters, we will use multiple boron agents with distinct intracellular distributions—BPA (boronophenylalanine), boric acid and BSH (sodium borocaptate). To assess the cell-structure parameters, we will employ cells with differing structural characteristics. The resulting survival data will be compared with LISMEC-predicted surviving fractions to evaluate the model’s validity.

In this study, we computed *D*_isoE_ with LISMEC in the radiation field at KUR; however, the precomputed data required by LISMEC are applicable to other neutron sources as well. By performing a macroscale radiation-transport simulation for the neutron source of interest and subsequently applying LISMEC to the simulation results, *D*_isoE_ can be computed using the same procedure. Therefore, the precomputed data developed in this work are generalizable.

This study clearly demonstrates the significant impact of microscopic parameters on the photon isoeffective dose (*D*_isoE_) in BNCT dosimetry, highlighting variations exceeding a factor of 4 under identical macroscopic boron concentrations. By utilizing LISMEC, microscale parameter analyses, which would conventionally require cumulative computational efforts spanning several months to potentially years if performed individually, can now be executed within seconds. In more detail, by using LISMEC, microdosimetry calculations that previously required 10 minutes per condition can now be completed in 1 second (600-fold speedup per condition). The precomputed database and linear interpolation approach employed by LISMEC provide rapid and efficient retrieval of the necessary microdosimetric parameters, greatly enhancing practical applicability in clinical settings. This evaluation framework, integrating the SMK model with LISMEC, facilitates rapid, flexible and accurate dose calculations, opening the way for personalized BNCT treatment planning.
