# Correction to: Development of Linear Interpolation System for SMK Model Parameters Evaluated from Cellular-Scale Simulation (LISMEC) and its application to BNCT dosimetry

**DOI:** 10.1093/jrr/rrag027

**Published:** 2026-04-14

This is a correction to: Takafumi Shigehira, Tubasa Watanabe, Minoru Suzuki, Yuho Hirata, Tatsuhiko Ogawa, Atsushi Fujimura, Yoshinori Sakurai, Tatsuhiko Sato, Development of Linear Interpolation System for SMK Model Parameters Evaluated from Cellular-Scale Simulation (LISMEC) and its application to BNCT dosimetry, *Journal of Radiation Research*, 2026; https://doi.org/10.1093/jrr/rraf075

The second name of the 6th co-author is corrected to read: “Atsushi Fujimura”.

